# Comparison of Immunomodulation Properties of Porcine Mesenchymal Stromal/Stem Cells Derived from the Bone Marrow, Adipose Tissue, and Dermal Skin Tissue

**DOI:** 10.1155/2016/9581350

**Published:** 2015-12-21

**Authors:** Sun-A Ock, Raghavendra Baregundi Subbarao, Yeon-Mi Lee, Jeong-Hyeon Lee, Ryoung-Hoon Jeon, Sung-Lim Lee, Ji Kwon Park, Sun-Chul Hwang, Gyu-Jin Rho

**Affiliations:** ^1^Department of Theriogenology and Biotechnology, College of Veterinary Medicine, Gyeongsang National University, 501 Jinju-daero, Jinju 660-701, Republic of Korea; ^2^Animal Biotechnology Division, National Institute of Animal Science & RDA, 77 Chuksan-gil, Kwonsun-Gu, Suwon 441-706, Republic of Korea; ^3^Department of Obstetrics and Gynecology, Institute of Health Science, School of Medicine, Gyeongsang National University, Jinju, Republic of Korea; ^4^Department of Orthopaedic Surgery, Institute of Health Science, School of Medicine, Gyeongsang National University, 501 Jinju-daero, Jinju 660-702, Republic of Korea; ^5^Research Institute of Life Sciences, Gyeongsang National University, 501 Jinju-daero, Jinju 660-701, Republic of Korea

## Abstract

Mesenchymal stromal/stem cells (MSCs) demonstrate immunomodulation capacity that has been implicated in the reduction of graft-versus-host disease. Accordingly, we herein investigated the capacity of MSCs derived from several tissue sources to modulate both proinflammatory (interferon [IFN] *γ* and tumor necrosis factor [TNF] *α*) and immunosuppressive cytokines (transforming growth factor [TGF] *β* and interleukin [IL] 10) employing xenogeneic human MSC-mixed lymphocyte reaction (MLR) test. Bone marrow-derived MSCs showed higher self-renewal capacity with relatively slow proliferation rate in contrast to adipose-derived MSCs which displayed higher proliferation rate. Except for the lipoprotein gene, there were no marked changes in osteogenesis- and adipogenesis-related genes following in vitro differentiation; however, the histological marker analysis revealed that adipose MSCs could be differentiated into both adipose and bone tissue. TGF*β* and IL10 were detected in adipose MSCs and bone marrow MSCs, respectively. However, skin-derived MSCs expressed both IFN*γ* and IL10, which may render them sensitive to immunomodulation. The xenogeneic human MLR test revealed that MSCs had a partial immunomodulation capacity, as proliferation of activated and resting peripheral blood mononuclear cells was not affected, but this did not differ among MSC sources. MSCs were not tumorigenic when introduced into immunodeficient mice. We concluded that the characteristics of MSCs are tissue source-dependent and their in vivo application requires more in-depth investigation regarding their precise immunomodulation capacities.

## 1. Introduction

Mesenchymal stromal/stem cells (MSCs) have significant clinical importance with their application not only in cell therapy for regenerative medicine and tissue engineering [1, 2], but also as immunomodulators that can reduce graft-versus-host disease (GvHD) that is associated with allografts and xenografts [3]. MSCs are found in various tissues, such as adipose tissue [4], bone marrow (BM) [5, 6], dermal tissue [7], synovial fluid [8], umbilical cord blood [9], and Wharton's jelly (WJ) [10], but their tissue-specific functional properties need an in-depth understandings.

There is growing interest in the use of stem cells in regenerative medicine, since they can be differentiated into a variety of cell types bone, adipose, cartilage, nerve myocardiocyte, and so forth upon exposure to signaling molecules and can be used to replace damaged cells. The critical features of stem cells are self-renewal, proliferative capacity, and differentiation potential. In comparison to pluripotent embryonic stem cells, many of these features are attenuated in multipotent MSCs, as they are derived from a somatic stem cell population [11]. The proliferative potential and self-renewal capacity of cells are directly related to telomerase activity and OCT3/4 expression [12, 13], respectively. In somatic stem cells, relative attenuation of these proliferation and self-renewal properties may be advantageous, as this may favor a low risk of tumorigenesis; however, this may be disadvantageous for cell therapy, where high regenerative capacity is required [11]. The relationship between these 2 properties has not been comprehensively studied against an isogenic background in MSCs derived from different tissues. Such information will be essential in order to develop suitable cell therapeutic methods based on MSCs.

MSCs have been reported to attenuate alloimmune responses due to their immunosuppressive capabilities in both innate and acquired responses in mice [14], humans [15], swine [16], and baboons [17]. This is likely due to their ability to secrete a variety of biologically active molecules, such as transforming growth factor (TGF), interleukin (IL) 10, and prostaglandin E2, each of which possesses immunomodulatory effects [3, 16]. However, most studies have focused on the immunomodulatory capability of BM-derived MSCs (BM-MSCs) in the treatment of GvHD induced by auto-, allo-, or xenografts [3, 18]. However, the immunomodulatory capability of MSCs derived from other tissues, such as adipose (A-MSCs) and dermal skin (DS-MSCs), is not completely understood.

Due to a lack of suitable in vitro or ex vivo models for studying most human diseases and the limitations of human organ construction in vitro, there is an increased demand for xenotransplantation and biomedical studies using animals. Rodents have been used to study a variety of human diseases, but they cannot recapitulate a number of key human physiological attributes and clinical symptoms of these diseases [19]. On the other hand pigs, with their anatomical, genetic, and pathophysiological similarities to humans, have been suggested as the best experimental model organism [20]. Therefore, immunological characterization of pig cells will be required for future xenotransplantation studies.

Accordingly, we designed the present study to investigate the correlation between self-renewal proliferation ability of different tissue-specific porcine MSCs followed by in vitro differentiation potential into osteocytes and adipocytes. We further evaluated immunotolerance properties by profiling proinflammatory (interferon [IFN] *γ* and tumor necrosis factor [TNF] *α*) and immunosuppression-related (TGF*β* and IL10) genes. Finally, we evaluated the tumorigenic propensity of these cells in vivo.

## 2. Materials and Methods

All animal samples were collected and handled following the approval of research ethical committee of Gyeongsang National University, animal center for biomedical experimentation under set guidelines (GNU-140305).

### 2.1. Chemicals and Media

Unless otherwise specified, all chemicals were purchased from Sigma Chemical Company (St. Louis, MO, USA) and media from Gibco (Invitrogen; Burlington, ON, Canada), respectively.

### 2.2. Cell Culture

For all experiments, micropigs (PWG Genetics, Seoul, South Korea) less than 1 month (*n* = 3) after birth were used for collecting adipose, bone marrow, and dermal skin tissues using standard surgical procedures. Adipose tissue-derived stem cells and bone marrow-derived stem cells were collected from abdomen and femur, respectively. A-MSCs were isolated from subcutaneous adipose tissue by using a previously described method [21], involving digestion with 0.075% collagenase type I, and were subsequently separated by filtration through 100- and 40-*μ*m cell strainers. BM-MSCs were isolated as previously reported [6]. DS-MSCs were isolated from the dermal layer of the ear skin, as described by Riekstina et al. (2008), after removing the epidermis. All cells were cultured in advanced Dulbecco's Modified Eagle Medium (ADMEM) supplemented with 10% fetal calf serum (FCS) and 1% penicillin-streptomycin (10,000 IU and 10,000 *μ*g/mL, resp.) at 38.5°C in a humidified atmosphere of 5% CO_2_, according to the method of Ock et al. (2010). Cells reached approximately 90% confluence at 7–10 days after being cultured (passage 0). Once confluency was achieved, cells were dissociated using 0.25% trypsin-ethylenediaminetetraacetic acid (EDTA) solution and pelleted at 300 ×g for 5 min. All further experiments were conducted in triplicate until otherwise specified.

### 2.3. Alkaline Phosphatase Activity (AP) Activity Assay

MSCs at passage 1 were grown on 35 mm *ϕ* dishes for 2 weeks and stained with an AP chromogen kit (BCIP/NBT; Abcam Inc.; Boston, MA, USA) to detect AP activity after being fixed with 4% formaldehyde. AP-positivity of MSCs was indicated by development of a purple-brown color.

### 2.4. In Vitro Differentiation Potential Assay

Cells from passage 3 at 80% confluence were induced to undergo adipogenic or osteogenic differentiation under specific culture conditions for 3 weeks. Briefly, for adipogenic differentiation ADMEM was supplemented with 10 *μ*M insulin, 200 *μ*M indomethacin, 500 *μ*M isobutylmethylxanthine, and 1 *μ*M dexamethasone, as reported by Vacanti et al. (2005). Differentiated adipocytes, after being fixed with 3.7% formalin, were stained with 0.5% oil red O solution for the detection of lipid droplets. Further, ADMEM supplemented with 1 *μ*M dexamethasone, 100 *μ*M ascorbic acid, and 10 mM *β*-glycerophosphate was used for osteogenic differentiation, as per the methods of Bosch et al. (2006). Formation of osteoblasts was analyzed by staining with 40 mM alizarin red after being fixed with 70% ethanol. The stained cells were washed several times with distilled water before being subjected to photography. The media was changed for every 3 days and the experiment was performed in triplicate.

### 2.5. Reverse Transcription-Polymerase Chain Reaction (RT-PCR) Assay

Total RNA was isolated from the MSCs before and after differentiation using the RNeasy Mini Kit (Qiagen, Valencia, CA, USA) in accordance with the manufacturer's instructions. cDNA synthesis for analyzing genes involved in adipogenesis (PPARG variant 2 [*PPARG*], lipoprotein A [*LPA*]), and in osteogenesis Runt-related transcription factor 2 [*RUNX*], osteocalcin [*BGLAP*], cell proliferation capacity [*TERT*], and stem cell renewal [*OCT4*], was performed from 1 *μ*g of total RNA, at 37°C for 60 min, using an Omniscript RT kit (Qiagen) with 10 *μ*M Oligo-dT primer (Invitrogen, Carlsbad, CA, USA). PCR reactions were performed in triplicate using the Maxime PCR pre-mix Kit (iNtRON BIO; Seongnam, Korea). Detailed information for each specific primer and associated PCR conditions is presented in Table 1. The glyceraldehyde-3-phosphate dehydrogenase gene (*GAPDH*) was used as a housekeeping gene for internal standardization. The PCR products were evaluated by electrophoresis on 1% agarose gel with 0.1 mg/mL ethidium bromide. Images were analyzed using zoom browser EX5.7 software (Canon, Tokyo, Japan).

### 2.6. Western Blot Assay

The western blotting was carried out using previously published protocol [23]; briefly cells at passage 3 were lysed with the nuclear and cytoplasm extraction reagent, RIPA buffer (Pierce Biotechnology, Rockford, IL, USA), and protein content was determined with the bicinchoninic acid (BCA) Protein Assay Reagent Kit (Pierce Biotechnology). HeLa whole cells and F9 cell lysate (Santa Cruz Biotechnology Inc.; Dallas, TX, USA) were used as positive controls for TERT and OCT3/4, respectively. Approximately 25 *μ*g of total protein was resolved on 12.5% sodium dodecyl sulfate-polyacrylamide gel electrophoresis (SDS-PAGE) and electroblotted onto polyvinylidene difluoride (Millipore; Billerica, MA, USA) membranes. The membranes were blocked and incubated with primary antibodies of anti-TERT (1 : 100 dilution; Santa Cruz Biotechnology, Inc.), anti-Oct3/4 (1 : 100 dilution; Santa Cruz Biotechnology Inc.), and anti*-β-*actin (1 : 1000 dilution; Cell Signaling Technology, Danvers, MA, USA) for overnight at 4°C, followed by incubation with horseradish peroxidase-conjugated donkey anti-goat (1 : 5000 dilution; OCT3/4 and TERT) or goat anti-rabbit (1 : 5000 dilution; *β*-actin) secondary antibodies (Santa Cruz Biotechnology Inc.) for 1 h at rt. The proteins were detected by immunoreactivity using an enhanced chemiluminescence kit (Amersham Biosciences, Little Chalfont, UK). *β*-actin was used to normalize protein loading.

### 2.7. Proliferation Assays

Cells at passage 3 were plated at 1000 cells per well of 24-well tissue culture plates (Thermo Scientific; Rockford, IL, USA) in triplicate. Cells from each well were detached by trypsinization and counted in duplicate using a hemocytometer, every 2 days for 14 days. The culture medium was changed every 3 days. Cell population doubling time was calculated using the formula DT = *t*(log⁡2)/(log⁡*Nt* − log⁡*No*), where *t* represents the culture time, and *No* and *Nt* are the initial and final cell numbers before and after seeding, respectively [6].

### 2.8. Flow Cytometry Assay

For analysis of CD29, CD45, CD90, and CD105 expression, in total, 1 × 10^6^ cells of all MSCs derived from 3 types of tissues at passage 3 were suspended in 100 *μ*L of Dulbecco's phosphate-buffered saline (DPBS) and labeled with fluorescein isothiocyanate- (FITC-) conjugated mouse antibodies CD45 (AbD Serotec, Raleigh, NC, USA), CD90 (BD Pharmingen, San Jose, CA USA), CD105 (Thermo Fisher Scientific Inc.), or Alexa-Fluor 647-conjugated antibody CD29 (BD Pharmingen). All antibody concentrations used for analysis of CD markers were adjusted to 10 *μ*g/mL. The data were analyzed for 10,000 cells/sample using BD FACS software (BD Biosciences, Franklin Lakes, CA, USA) and were compared to isotype-matched controls.

For analysis of the cell cycle, cells (5 × 10^3^ cells/sample) were fixed with 70% ethanol, washed twice in DPBS, and stained with 10 *μ*g/mL propidium iodide solution for 30 min. DNA content of the each cell was measured and categorized as G0/G1, S, or G2/M phase of the cell cycle.

For analysis of immunologic tolerance-related proteins, such as IFN*γ* (an immunoregulatory and proinflammatory molecule), TNF*α* (an inflammatory cytokine), TGF*β*1/2 (inhibitor of T cell proliferation), and IL10 (an anti-inflammatory cytokine), cells (1 × 10^4^ cells/sample) at 90% confluence were recovered by trypsinization and stained with FITC-conjugated mouse monoclonal IgG1 for IFN*γ* and TNF*α* (Santa Cruz Biotechnology Inc.), unconjugated monoclonal antibodies such as rat monoclonal IgG2a and mouse monoclonal IgG1 for TGF*β*1/2 and IL10, respectively (Santa Cruz Biotechnology Inc.), or with isotype-matched controls, according to the manufacturer's instructions. In addition, TGF*β*1/2 and IL10 unconjugated antibodies were detected by 30 min incubation with either goat anti-rat IgG FITC (Santa Cruz Biotechnology Inc.) or goat anti-mouse IgG FITC (Santa Cruz Biotechnology Inc.) secondary antibodies, respectively.

Analysis of OCT3/4 expression was performed using 5 × 10^3^ cells/sample according to the manufacturer's instructions (STEMCELL Technologies; Vancouver, Canada). Analyses of the cell cycle and the expression of OCT3/4 and immunologic tolerance-related proteins were performed in triplicate using a flow cytometer (Becton Dickinson FACSCalibur; Franklin Lakes, NJ, USA) at passage 3.

### 2.9. In Vivo Teratoma Formation Assay

MSCs (1 × 10^7^ cells/mL) harvested by trypsinization were labeled with 2 × 10^−6^ M PKH26 red fluorescent cell linker for 5 min. Labeled MSCs were resuspended in Ca^2+^-free DPBS incorporating 30% reduced Matrigel (M; BD Biosciences; Franklin Lakes, NJ, USA), maintained on ice, and drawn into a 1-mL syringe immediately before injection. Subcutaneous injection of MSCs into mice was performed according to the method of Prokhorova et al. (2009) [24]. Overall 18 male NOD.CB17-Prkdc^scid^ mice (3 mice/group) and 3 male ICR mice (Charles River Laboratories Inc., Wilmington, MA, USA) (for the MDA-MB-231 group) of 6-week-old were obtained from the Western Australia (Canning Vale, Australia) and Japan Animal Resources Centers (Shizuoka, Japan). Experimental groups were divided into control group (no treatment), PBS + M, MDA-MB-231 (ATCC; positive control) + M, A-MSCs + M, BM-MSCs + M, and DS-MSCs + M. Approximately 1-2 × 10^6^ cells in 200 *μ*L/injection were injected into the dorsolateral area at the subcutaneous space on both sides of male NOD.CB17-Prkdc^scid^ mice and male ICR mice. After 9 weeks, when the tumor diameters reached 1.5 cm, the mice were sacrificed by cervical dislocation and tumors were surgically recovered. For histological analysis, tumors were fixed with 3.7% formalin for 1 day, dehydrated with 20% sucrose solution for 1 day, and embedded with OCT compound (Tissue-Tek; Tokyo, Japan) on LN_2_ for cryosectioning. Sections were cut to a thickness of 10 *μ*m. Histochemical staining was performed with hematoxylin and eosin (H&E) solution. Immunofluorescence staining was performed with 1 *μ*g/mL 4′,6-diaminido-2-phenylindole solution as a counterstain for 30 min. The samples were observed under a fluorescence microscope (Leica; Wetzlar, Germany).

### 2.10. Xenogeneic MSC-Mixed Lymphocyte Reaction (MLR)

MLR was performed with each MSC line derived from pigs (*n* = 3) (less than 1 month old) in triplicate [25–27]. In advance, MSCs used for MLR were identified using both CD mark analysis and in vitro differentiation capacity (adipogenesis and osteogenesis), as described above. Verified MSCs (1 × 10^4^ or 1 × 10^5^ cells/sample) in 200-*μ*L culture medium were seeded in standard 96-well plates. Human peripheral blood monocytes (PBMCs, 1 × 10^5^ cells/sample) purchased from Cellular Technologies (Shaker Heights, OH, USA) were added directly to the MSCs. PBMCs were either resting or activated with 5 *μ*g/mL concanavalin A (ConA). Resting or activated PBMCs or MSCs alone served as controls. Tissue culture plates were incubated under 5% CO_2_ in an incubator for 4 days. Cell proliferation was assessed by enzyme-linked immunosorbent assays (ELISA) using a Cell Proliferation ELISA BrdU (colorimetric) kit (Roche, Mannheim, Germany) in accordance with the manufacturer's recommendations. BrdU was added for the last 24 h of the culture period.

### 2.11. Statistical Analysis

The statistical significance of differences among groups was analyzed using one-way analysis of variance (ANOVA) with SPSS 12.0 (SPSS Inc.; Chicago, IL, USA) followed by Tukey's or least-square difference (LSD) multiple comparisons tests. Values are expressed as mean ± standard error (SEM). Differences were considered to be significant when *P* < 0.05.

## 3. Results

### 3.1. Generation of MSCs

**Figure 1:**
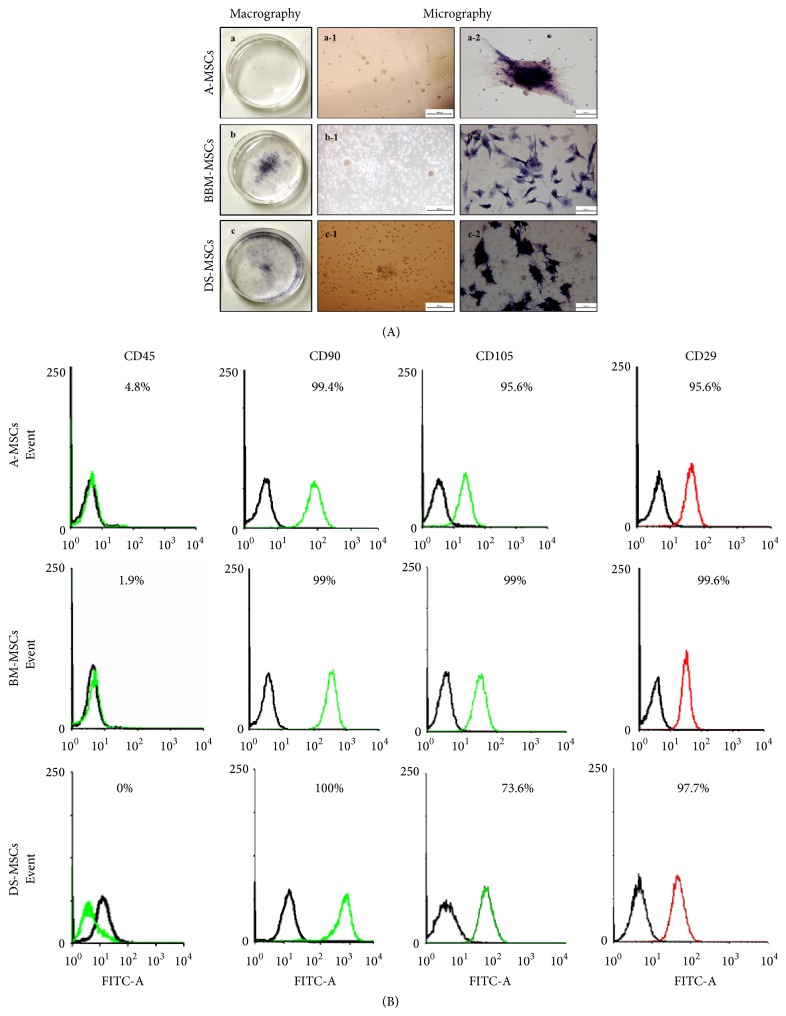
Analysis of alkaline phosphatase (AP) and CD marks in mesenchymal stromal/stem cells (MSCs). For analysis of AP activity, each type of passage 1 MSCs was cultured until ca. 80% confluence on 35 mm dishes, fixed in 3.7% formalin, and stained with a BCIP/NBT kit (A). a, b, and c show A-MSCs, BM-MSCs, and DS-MSCs, respectively, derived from a female micropig. MSCs were observed by both macrography (a–c) after AP staining and micrography before (a-1, b-1, and c-1; scale bars = 500 *μ*m) and after (a-2, b-2, and c-2; scale bars = 200 *μ*m) AP staining. The presence of purple/brown color on AP staining was judged to be a positive reaction. (B) MSC-positive (CD90, 105, and 29) and -negative (CD45) CD marks measured in 1 × 10^5^ cells per sample by flow cytometry. Open black histogram represents the isotype-matched control, and green and red open histograms represent positive CD marks.

As shown in Figure 1(A), MSCs from adipose, bone marrow, and dermal tissues of 1-week-old micropigs were successfully isolated and cultured. The cells exhibited a fibroblast-like morphology and MSCs from all tissue types were able to form colonies. AP activity was observed in MSCs from all tissues, although the absolute level of staining varied; the lowest level was observed in A-MSCs.

As shown in Figure 1(B), CD45, as a hematopoietic stem cell marker, was virtually not observed in any of the MSC types, and CD90, CD105, and CD29, as an MSC-positive marker, were expressed in more than 90% of all MSC types, except for CD105, whose expression was 73.6% in DS-MSCs. Therefore, cells derived from 3 types of tissues were confirmed to possess MSC characteristics.

### 3.2. In Vitro Differentiation of MSCs

**Figure 2:**
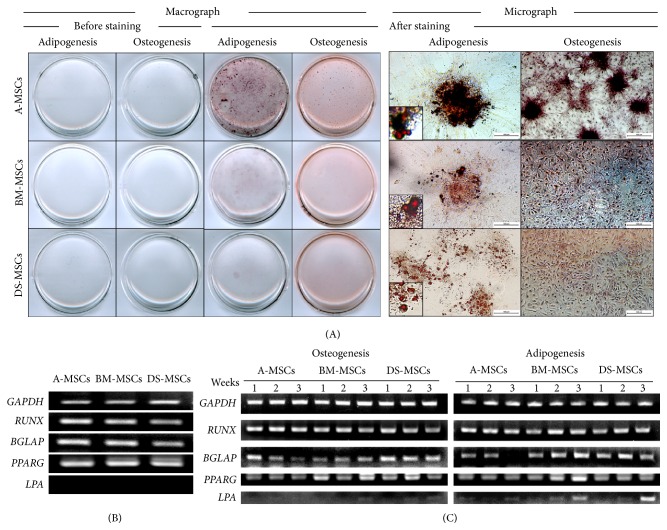
In vitro differentiation of passage 3 mesenchymal stromal/stem cells (MSCs). MSCs at ca. 80% confluence were subjected to osteogenic or adipogenic conditions, and differentiation was confirmed by staining with alizarin red S or oil red O solution, respectively. (A) MSCs were observed by both macrography before and after differentiation and micrography after differentiation showing A-MSCs, BM-MSCs, and DS-MSCs, respectively, derived from a female micropig. Scale bars = 200 *μ*m. MSCs were analyzed for the expression of osteogenesis- (*RUNX2* and* BGLAP*) and adipogenesis- (*PPARG* and* LPA*) related genes before (B) and at 1-week intervals after (C) differentiation. The housekeeping gene* GAPDH* was used as a control, shown in (B) and (C).

Tissue-specific MSCs at passage 3 were differentiated into adipocytes or osteocytes in specific media for 3 weeks, as shown in Figure 2. Cells were then analyzed using histochemistry (Figure 2(A)) and RT-PCR (Figure 2(B)). Histochemistry confirmed that all specific tissue MSC types underwent differentiation into adipocytes and osteocytes, as confirmed by oil red O and alizarin red S staining, respectively. The macrograph results indicated that A-MSCs exhibited the highest capacity to differentiate into both adipocytes and osteocytes.

Osteogenesis-related mRNAs,* RUNX* and* BGLAP*, and adipogenesis-related mRNAs,* PPARG* and* LPA*, were analyzed using RT-PCR over the 3-week differentiation period (Figures 2(B) and 2(C)).* RUNX*,* BGLAP*, and* PPARG* were expressed in all MSC types prior to differentiation, but* LPA* was absent (Figure 2(B)). The levels of* RUNX* did not change following osteogenic or adipogenic differentiation into MSCs derived from any tissue.* BGLAP* expression increased at 1 week after osteogenesis in A-MSCs and decreased by 3 weeks, whereas, in BM-MSCs, the level was constant for up to 3 weeks, and in DS-MSCs, it increased at 1 week and was maintained for up to 3 weeks. The reduction in* BGLAP* after adipogenesis was observed only in A-MSCs at 3 weeks, whereas it remained constant in all other MSC types. There was no difference in the expression of* PPARG* after adipogenesis in A-MSCs and DS-MSCs, but its levels increased in BM-MSCs for up to 3 weeks. There was no reduction in* PPARG* levels after osteogenesis in any MSC type.* LPA* was expressed in all MSC types after adipogenesis and, in particular, BM-MSCs and DS-MSCs at 3 weeks. However, there was no change in* LPA* expression after osteogenic differentiation in any MSC type.

### 3.3. Proliferation of MSCs

**Figure 3:**
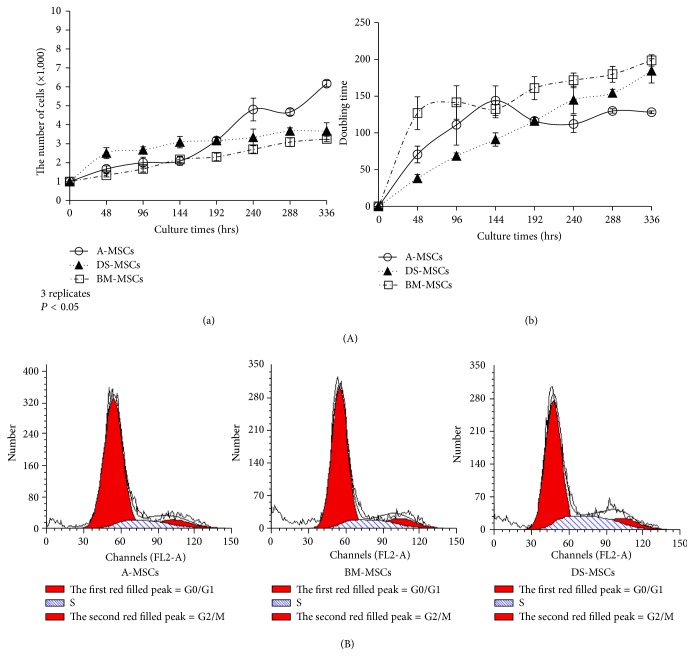
In vitro proliferation capability of passage 3 mesenchymal stromal/stem cells (MSCs). (A) and (B) display growth rate (absolute growth rate and doubling time in a & b, resp.) parameters of MSCs and the cell cycle, respectively. For analysis of the growth rate, MSCs were seeded at 1 × 10^3^ cells/well in 24-well tissue culture plates, and the number of cells was calculated at 48 h intervals. For cell cycle analysis, MSCs were seeded at a density of 1 × 10^5^ cells per 35 mm dish, cultured until ca. 80% confluence, and stained with propidium iodide (PI). The first red line, the second blue oblique lines, and the third red-filled peak indicate the G0/G1, S, and G2/M phases, respectively. Experiments shown in (A) and (B) were performed in triplicate.

To analyze the proliferative capacity of MSCs, we estimated doubling time at an interval of 2 days, as shown in Figures 3(A) and 3(B). DS-MSCs (3.1 ± 0.5) showed the highest growth rate for up to 144 h when compared to A-MSCs (2.1 ± 0.4) and BM-MSCs (2.2 ± 0.3). A-MSCs (4.8 ± 1.0) exceeded DS-MSCs (3.3 ± 0.8) at 240 h, and A-MSCs (6.2 ± 0.3) maintained the highest growth rate among all MSC types for up to 336 h (Figure 3(A)). Doubling time, based on the results shown in Figure 3(A), was calculated as shown in Figure 3(B). This was comparable between DS-MSCs (91.1 ± 15.6 h) and A-MSCs (143.8 ± 20.2 h), whereas BM-MSCs (132.8 ± 34.7 h) had the shortest doubling time for up to 144 h. A-MSCs (129.8 ± 17.4) began to decrease earlier than DS-MSCs (144.8 ± 8.6) at 240 h, and this decrease continued for up to 336 h.

The results of the cell cycle analysis are shown in Figure 3(B) and Table 2. As shown in Table 2, the percentage of cells in G0/G1 was significantly lower (*P* < 0.05) in DS-MSCs (67.8 ± 2.9) than in A-MSCs (77.9 ± 2.8) and BM-MSCs (77.4 ± 2.5). There were no significant differences in the S phase fraction between DS-MSCs (16.8 ± 3.5) and BM-MSCs (14.2 ± 1.9), although DS-MSCs had the highest overall percentage of any MSC type.

### 3.4. OCT3/4 and TERT Expression in MSCs

**Figure 4:**
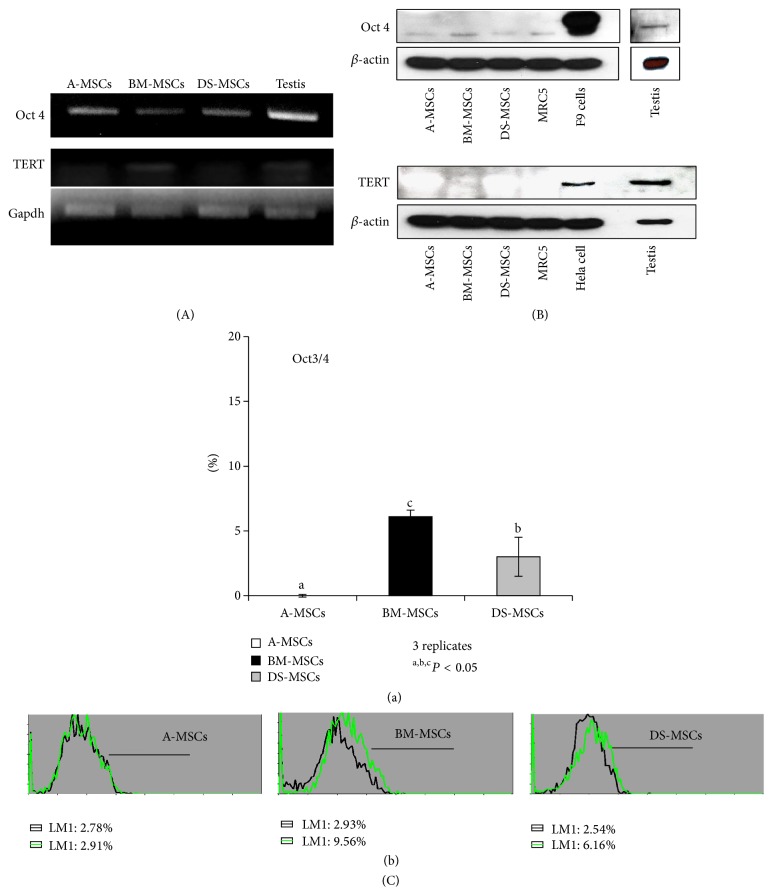
Analysis of OCT3/4 and TERT in passage 3 mesenchymal stromal/stem cells (MSCs). (A) RT-PCR for* OCT4* and* TERT*. (B) Western blot of OCT3/4 and TERT. Porcine testis and MRC5 cells were used as positive control and negative control for both* OCT4* and* TERT*, respectively. F9 and HeLa cells were used as positive controls for OCT3/4 and TERT, respectively. The internal control was* GAPDH* for RT-PCR and *β*-actin for western blot. (C) Protein expression of OCT3/4, measured by flow cytometry, was performed in triplicate ((C)a). ^a, b, c^ *P* < 0.05. ((C)b) black and green open histograms indicate negative and positive immunoreactivity, respectively.

We next analyzed* OCT3/4* and* TERT* mRNA and protein expression, as shown in Figure 4. Porcine testis tissue was used as a species-specific positive control for monitoring the mRNA and protein expression of* OCT3/4* and* TERT*. MRC5, F9, and HeLa cells were used as normal human control cells,* OCT3/4*-positive controls, and* TERT*-positive controls, respectively. Expression of* OCT4* was detected in all MSC types (Figure 4(A)), whereas* TERT* was only detected weakly in BM-MSCs. With respect to protein expression (Figure 4(B)), TERT was not detected in any MSC type. Flow cytometry revealed that OCT3/4 expression (Figure 4(C)) was significantly higher in BM-MSCs (6.1 ± 0.5) than in DS-MSCs (3.0 ± 1.5) and A-MSCs (0 ± 0.1), although the absolute level of OCT3/4 was low (Figure 4(C)a).

### 3.5. Expression of Immunomodulation-Related Proteins

**Figure 5:**
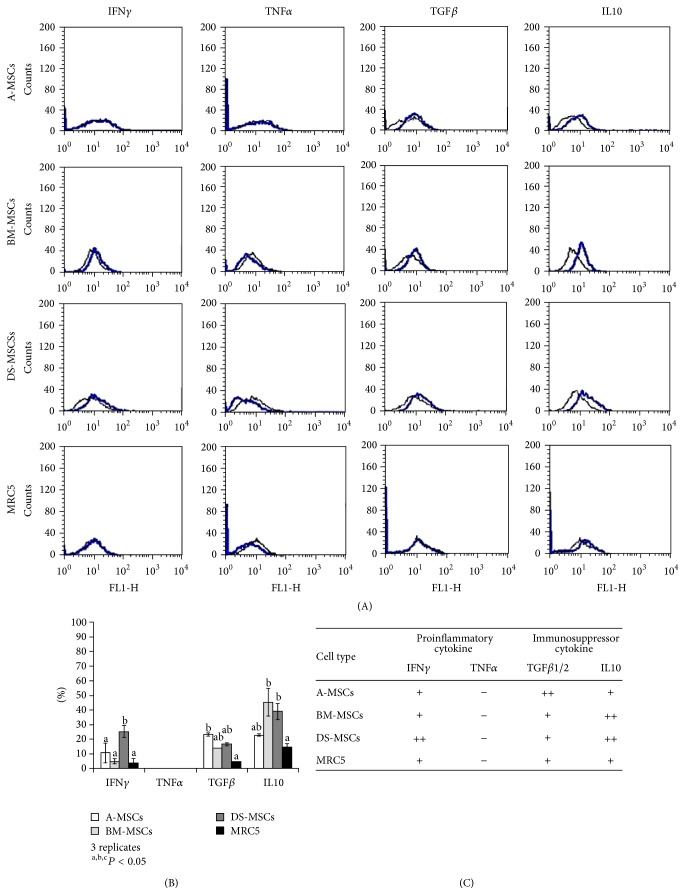
Expression of immunomodulators in passage 3 mesenchymal stromal/stem cells (MSCs). (A) Histograms of the proinflammatory cytokines IFN*γ* and TNF*α* and the immunosuppressive cytokines TGF*β*1/2 and IL10 (black, isotype-matched control; blue, positive controls). MRC5 cells were used as negative control for all cytokines. This experiment was performed in triplicate. (B) Expression rate (%). (C) Cytokine secretion based on the results of MRC5 cells in (B) displayed as falling, + (expression), or − (no expression) signs. ++ indicates a significant increase relative to MRC5.

The results of analysis of the immunomodulatory capabilities of MSCs are shown in Figure 5. Expression of IFN*γ* was significantly higher in DS-MSCs (25.3 ± 4.0) than in A-MSCs (10.7 ± 6.7), BM-MSCs (5 ± 1.7), and MRC5 cells (3.7 ± 3.2). TNF*α* was not detected in any of the MSC types. The expression of TGF*β*1/2 was significantly higher in A-MSCs (23.3 ± 0.6) than in MRC5 cells (4.7 ± 2.5) but was not significantly different from that in BM-MSCs (14.0 ± 9.5) and DS-MSCs (16.7 ± 5.7). IL10 levels were significantly higher in both BM-MSCs (45.3 ± 12.4) and DS-MSCs (39.0 ± 9.5) than in MRC5 cells (14.7 ± 7.2) but were not significantly different from those in A-MSCs (23 ± 5.6). Compared to the expression profile in MRC5 cells, there was a significant increase in cytokine levels in MSCs, as displayed in Figure 5(C). IFN*γ* levels only increased in DS-MSCs, whereas TGF*β*1/2 levels increased in A-MSCs and IL10 levels were elevated in both BM-MSCs and DS-MSCs.

### 3.6. Subcutaneous Teratoma Formation

**Figure 6:**
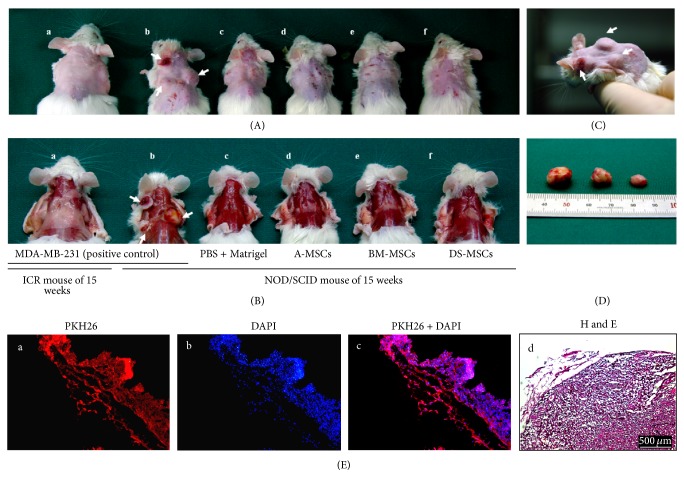
Tumor formation in mesenchymal stromal/stem cells (MSCs) transplanted into immunodeficient mice. (A) and (B) Images obtained after elimination of dorsal hair or clothing, respectively, in each mouse. (a–f) Cells (1 × 10^7^) stained with PKH26 were transplanted to both dorsal subcutaneous spaces of ICR mice (a) or NOD/SCID mice (b–f), and the mice were sacrificed after 15 weeks to confirm tumor formation. ((A)a-b) and ((B)a-b) show mice injected with MDA-MB231 as a positive control and c–f show mice injected with PBS + Matrigel (M), A-MSCs + M, BM-MSCs + M, and DS-MSCs + M, respectively. (C) and (D) display tumors isolated from mouse (A)b (Figures 6(A)-6(b)). ((E)a–c) Immunohistochemical staining and ((E)d) H&E staining were performed in tissue sections from tumors. ((E)a) and ((E)b) Red and blue identify the PKH26-stained membrane of cells and counterstaining of nucleic acids, respectively. Arrows indicate the blood vessels. Scale bars = 500 *μ*m.

NOD.CB17-Prkdc^scid^ mice were then used for analysis of in vivo teratoma formation. At 4 weeks after transplantation, 2 NOD.CB17-Prkdc^scid^ mice injected with MDA-MB-231 did not demonstrate any teratoma formation. At 5 weeks after transplantation, 1 NOD.CB17-Prkdc^scid^ mouse injected with MDA-MB-231 had small (2-3 mm) tumors that were not observed in other mice in the group. At 9 weeks after transplantation, tumors from NOD.CB17-Prkdc^scid^ mice injected with MDA-MB-231 exceeded 15 mm in diameter (Figure 6); all mice were then sacrificed to analyze tumor size in detail (Figures 6(A) and 6(B)). Tumors on the right flank were 15 mm in diameter and tumors on the left flank were divided into 2 groups of 9 mm and 7 mm diameters (Figure 6(D)). No teratomas were observed in mice injected with PBS or MSCs.

Tumors had PKH26-positive membranes (red) around the nucleus (Figure 6(E)), confirming that the cells originated from the injected MDA-MB-231 cells. Blood vessels (arrows) surrounding the tumors were detected by H&E staining (Figure 6(E)).

### 3.7. Xenogeneic MSC-MLR

**Figure 7:**
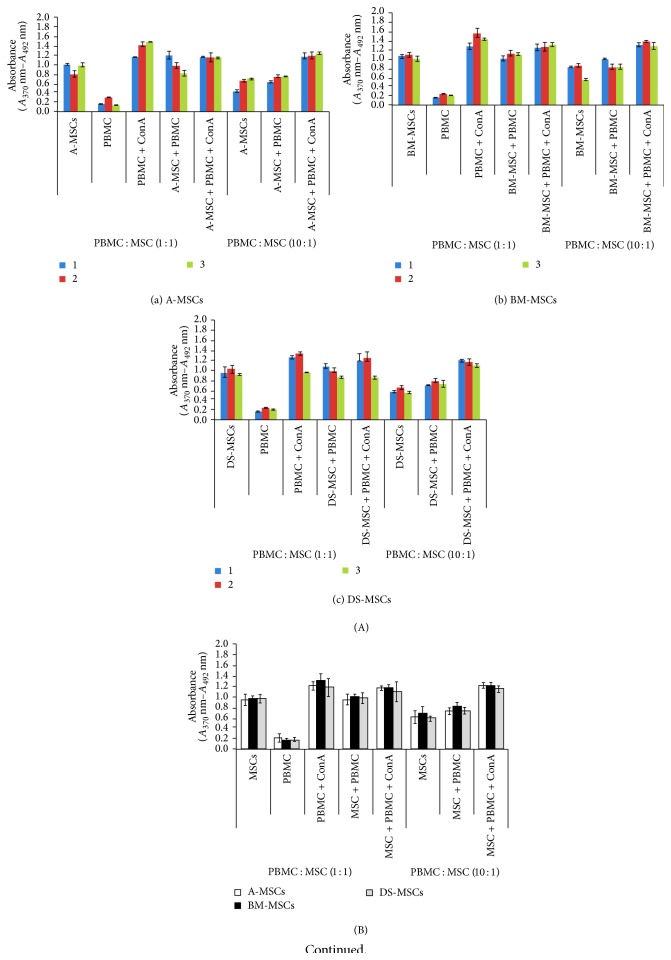
Xenogeneic human mixed lymphocyte reaction (MLR) test to confirm immunomodulation capacity of mesenchymal stromal/stem cells (MSCs). Tissue-specific MSCs were isolated from 3 pigs. Peripheral blood mononuclear cells (PBMCs) were cocultured with MSCs in 2 ratios (PBMCs : MSCs of 1 : 1 and 10 : 1). (A) and (B) show the result of the MLR test for each MSC type, by MSC source, based on the average value obtained from the 3 pigs. (C) Cell states at 72 h after cell seeding on 96-well plates. Scale bars = 500 *μ*m.

As shown in Figure 7, an MLR test was performed to confirm the immunomodulation capacity of MSCs among tissue-specific MSC types derived from pigs (*n* = 3). PBMCs were treated with (ConA + PBMC, positive control) or without ConA (PBMC) and cocultured with MSCs (ratio of PBMCs : MSCs = 1 : 1 and 10 : 1). We aimed to determine whether MSCs can reduce proliferation of PBMCs in the presence of ConA (ConA + PBMC). Regardless of the ratio of PBMCs : MSCs, there was a difference in the average proliferation between ConA + PBMCs and ConA + PBMCs + MSCs among pigs (Figure 7(A)), but there was no difference among tissue-specific MSC types (Figure 7(B)). In the case of PBMC + MSCs (PBMCs : MSCs, 1 : 1 and 10 : 1), MSCs did not increase the number of resting PBMCs, regardless of the MSC types. Therefore, the present study revealed that MSCs did not reduce proliferation of activated PBMCs and the immunomodulation capacity did not differ among tissue-specific MSCs on the basis of the MLR test.

## 4. Discussion

Here, we have identified a relationship between the self-renewal and proliferative potential in tissue-specific MSC types in vitro. Furthermore, our in vivo experiments revealed that there was no risk of teratoma when MSCs were transplanted into immunodeficient mice. In addition, the potential of MSCs to reduce GvHD was compared using immunomodulatory markers as a proxy among tissue-specific MSC types. This confirmed that BM-MSCs and A-MSCs are likely to be more potent immune modulators than are DS-MSCs.

The present study used 3 kinds of MSCs extracted from specific tissues under the same genetic conditions; all the MSCs showed a fibroblast-like morphology and the ability to form colonies in vitro. Although we did not directly compare the number of colonies among specific tissue MSC types, A-MSCs tended to form colonies more efficiently when compared with the others. This result was expected, since A-MSCs have greater proliferative potential than the other MSC types.

Contradictory results have been obtained in studies of tissue-specific MSCs using AP activity as a measure of stem cell maintenance capability [28]. Specifically, although A-MSCs were more capable of undergoing in vitro differentiation, they also had the lowest AP activity. Consistent with this, we also found that A-MSCs have extremely low AP activity but have a higher potential for differentiation along the osteogenesis and adipogenesis pathways, than do other MSC types, although our studies were performed with canine cells [29]. Therefore, we inferred that A-MSCs may be more vulnerable in terms of maintenance of stem cells than BM-MSCs and DS-MSCs. In addition, based on CD mark analysis, all tissue-specific cells were recognized as MSCs, because expression of CD29, -90, and -105, as MSC-positive markers under plate culture conditions, was observed [6].

We obtained further information regarding the differentiation capacity of MCSs by performing molecular profiling. All undifferentiated MSC types expressed the osteogenesis-related genes* RUNX2* and* BGLAP* and the adipogenesis-related gene* PPARG*. Previous studies have reported similar results in different species [29, 30]. The similar expression profile is presumably due to the common mesodermal origin of MSCs. When osteogenesis was induced, the expression of* RUNX2*, a transcription factor associated with osteoblast differentiation, was maintained in all MSC types, and* BGLAP* levels increased in DS-MSCs. When adipogenesis was induced,* BGLAP* was not induced in A-MSCs, whereas* PPARG* increased in BM-MSCs and* LPA* increased in all MSC types. In summary, the mRNA profiling did not reveal a common osteogenic or adipogenic marker that provided absolute correlation with differentiation potential. The reason for this might be the low levels of sensitivity. Alternatively, each MSC type may activate a unique subset of genes that together converge to enforce differentiation. Future studies will need to examine a broader panel of genes to address this question.

In terms of regenerative medicine, the limited proliferative capacity of MSCs is a major bottleneck that must be overcome before these cells can be used therapeutically. For example, the fraction of BM-MSCs is markedly reduced with aging in humans [3], and prolonged cell culture in vitro induces rapid aging of porcine MSCs [31]. Eight days after seeding, the growth rate of A-MSCs was significantly higher than that of other cell types; however, the proportion of cells in G0/G1 was the lowest in DS-MSCs. The underlying cause of these differences was investigated by determining the growth rate and cell cycle using different seeding concentrations (1 × 10^3^ and 1 × 10^5^ cells) and culture durations (14 days and 4-5 days). A previous report noted that the growth rate of A-MSCs was faster than that in BM-MSCs during short-term culture in vitro [32, 33]. On the other hand, after long-term in vitro culture for more than 60 days, the expansion potential was lost in A-MSCs but was retained in BM-MSCs [34]. The limited long-term proliferation potential of A-MSCs may reduce the chance of premalignant proliferation. This has obvious positive safety attributes and therefore A-MSCs may be the most suitable cell type for clinical applications.

A key factor in the maintenance of stem cell properties is the transcription factor OCT3/4; indeed, insertion of* OCT4* into somatic cells facilitates their conversion to pluripotency [35]. Most reports have indicated that OCT4 expression in somatic stem cells depends on cell passage number, cell source, and age [6, 28, 36]. Here, we detected both* OCT4* mRNA and protein in all MSC types. The OCT3/4 expression was confirmed by flow cytometry in DS-MSCs and BM-MSCs; its expression level was low in all MSC types; however, among them BM-MSCs depicted the highest levels of OCT3/4. We therefore inferred that BM-MSCs retain a stronger stem cell-like capacity than do other MSC types.

The proliferative potential of MSCs for the clinical utility could be predicted by analysis of telomerase activity [37]. There have been conflicting reports regarding the expression of telomerase in MSCs, likely due to the sensitivity of measurements and the lack of appropriate standards. In general, the level of telomerase expression in MSCs is thought to be very low [37–41]. In the present study, we observed* TERT* mRNA expression exclusively in BM-MSCs, although the protein was not detected. Therefore, we concluded that TERT expression is extremely low in ex vivo cultured MSCs. BM-derived MSCs may exhibit a prolonged life span in vitro, compared to those derived from adipose tissue and dermal skin. In addition, our results revealed that the level of telomere activity in MSCs varies in a tissue-specific manner.

Recently, immunosuppressive capacities, such as inhibitory effects on T, B, dendritic, and natural killer cell proliferation, have been demonstrated for MSCs [42]. This is likely because MSCs can secrete a variety of bioactive molecules, including PGE2, TGF*β*, and IL10, and because the immunosuppressive capabilities of MSCs are underscored by their ability to reduce the risk of GvHD in allografts [3, 16, 43]. The present study revealed that both A-MSCs and BM-MSCs are probably capable of immunosuppressive activities. In contrast, DS-MSCs coexpress IFN*γ* and IL10, likely attenuating their immune regulatory functions as compared to other MSC types. Thus, the immunosuppressive abilities of MSCs are probably dependent on the tissue source from which they are derived.

ES cells form teratocarcinomas in transplanted organs in vivo due to their high and inordinate proliferation and differentiation capability, which limits their clinical application [44, 45]. Thus, it is critical to determine whether MSCs exhibit this tendency in vivo. Recently, in vivo systemic immunosuppression favoring tumor growth as a side effect of MSCs has been reported [46–48]. Thus, treatment with MSCs could be dangerous in patients with preexisting malignant conditions. Reassuringly, we did not find any evidence of MSC-derived teratomas following in vivo injection. However, cell-based therapies that incorporate MSCs should still be strictly monitored. This is particularly true for BM-MSCs, in which low but detectable telomerase activity was observed.

Finally, the present study compared the immunomodulation capacity among tissue-specific MSCs derived from 3 pigs and revealed that tissue-specific MSCs did not induce an increase of activated or resting PBMCs, and activated PBMCs did not attack MSCs. Therefore, tissue-specific MSCs were shown to have a similar, partial immunomodulating capacity, as the MLR test results did not differ among the tissue-specific MSCs. However, the data from the few available pig xenogeneic MSC-MLR tests are conflicting [25, 26]. The reason for the different findings with regard to the immunomodulating capacity of MSCs may be due to use of the *t*-test method, breed, age, differences in species used, and so forth [25, 26, 49].

The present study revealed an unexpected mismatch between the self-renewal and proliferative capacities of MSCs cultured in vitro. We also showed that tissue-specific MSCs may retain characteristics of their original tissue source in terms of their differentiation capability and specific cytokine gene expression profile. In the case of BM-MSCs, self-renewal capacity was high, but proliferative capacity was low, whereas the converse was true for A-MSCs. The immunosuppressive capacity, as determined by cytokine gene expression profile of both A-MSCs and BM-MSCs, was superior to that of DS-MSCs. However, the immunomodulation capacity as determined by xenogeneic MLR test did not differ among MSCs from different sources. Therefore, a more detailed comparison of the immunomodulation capacity, using tissue-specific MSCs after in vivo xenogeneic transplantation of MSCs, should be made in future.

## 5. Conclusion

We concluded that the characteristics of MSCs are tissue source-dependent however with our limited experimental results it is not possible to establish which cell source is optimal for clinical or preclinical treatments involving MSCs. Present study also revealed that A-MSCs are likely to have the most beneficial effects in short-term treatment regimens given their low telomerase activity and rapid proliferation rate. Although they appear to be relatively safe for in vivo use, more details regarding their precise immunomodulation capacities are required in order to optimize their clinical use.

## Figures and Tables

**Table 1:** Primer sequence for gene expression analysis.

Genes	Sequence 5′-3′	Products sizes (bP)	Annealing temp. (°C)	Accession number or reference	Cycling numbers
Peroxisome proliferator-activated receptor gamma 2 (PPAR*γ*2)	F′-GCGCCCTGGCAAAGCACT	238	63	AF103946	35
	R′-TCCACGGAGCGAAACTGACAC				

Lipoprotein Lipase (LPL)	F′-GCAGGAAGTCTGACCAATAAG	183	55	Qu et al. [22]	35
	R′-GGTTTCTGGATGCCAATAC′				

Sus scrofa Runt-related transcription factor 2 (Runx2)	F′-GCTCTTCCCAAAGCCAGAG	205	60	EU668154	35
	R′-TTGTCAACGCCATCGTTCT				

Osteocalcin (OC)	F′-TCAACCCCGACTGCGACGAG	165	55	AW346755	35
	R′-TTGGAGCAGCTGGGATGATGG				

Telomerase Reverse Transcriptase (TERT)	F′-TGCTCGCCAACGTTTACA	117	52	AY785158	35
	R′-CAAGCCGGAGGAAAAATG				

Octamer-Binding transcription factor 4 (Oct4)	F′-AGGTGTTCAGCCAAACGACC	335	60	AJ251914	35
	R′-TGATCGTTTGCCCTTCTGGC				

Glyceraldehydes-3-phosphate dehydrogenase (Gapdh)	F′-TCGACCACAGGGTAGGTTTC	497	45	AF017079	35
	R′-CCCCAGCATCAAAGGTAGAA				

**Table 2:** Cell cycle of MSCs derived from fat, bone marrow and dermal ear skin of a micropig 3 replicates *P* < 0.05.

Groups	Cell cycle (Mean ± SD)		
	GO/G1	S	G2/M
A-MSCs	77.9 ± 2.8^b^	7.3 ± 5.0^a^	14.8 ± 4.7
BM-MSCs	77.4 ± 2.5^b^	14.2 ± 1.9^ab^	8.5 ± 2.0
DS-MSCs	67.8 ± 2.9^a^	16.8 ± 3.5^b^	15.5 ± 5.6

The different superscript (a, b, ab) indicate significant (*P* < 0.05) differences among MSCs types.
